# Adequacy of maternal anesthesia depth with two sodium thiopental doses in elective caesarean section: a randomized clinical trial

**DOI:** 10.1186/s12871-021-01421-7

**Published:** 2021-08-10

**Authors:** Golnar Sabetian, Farid Zand, Fatemeh Mirhadi, Mohammad Reza Hadavi, Elham Asadpour, Laleh Dehghanpisheh, Zeinabsadat Fattahi Saravi, Seyed Mostajab Razavi

**Affiliations:** 1 Trauma Research Center, Shiraz University of Medical Sciences, Shiraz, Iran; 2 Anesthesiology and Critical Care Research Center, Shiraz University of Medical Sciences, Shiraz, Iran; 3 Neonatal Research Center, Shiraz University of Medical Sciences, Shiraz, Iran

**Keywords:** General anesthesia, Apgar score, Cesarean section, Newborn, Thiopental

## Abstract

**Background:**

Administration of an optimal dose of anesthetic agent to ensure adequate depth of hypnosis with the lowest risk of adverse effects to the fetus is highly important in cesarean section. Sodium thiopental (STP) is still the first choice for induction of anesthesia in some countries for this obstetric surgery. We aimed to compare two doses of STP with regarding the depth of anesthesia and the condition of newborn infants.

**Methods:**

In this clinical trial, parturient undergoing elective Caesarian section were randomized into two groups receiving either low-dose (5 mg/kg) or high-dose (7 mg/kg) STP. Muscle relaxation was provided with succinylcholine 2 mg/kg and anesthesia was maintained with O2/N2O and sevoflurane. The depth of anesthesia was evaluated using isolated forearm technique (IFT) and bispectral index (BIS) in various phases. Additionally, infants were assessed using Apgar score and neurobehavioral test.

**Results:**

Forty parturient were evaluated in each group. BIS was significantly lower in high-dose group at skin incision to delivery and subcutaneous and skin closure. Also, significant differences were noticed in IFT over induction to incision and incision to delivery. Apgar score was significantly lower in high-dose group at 1 min after delivery. Newborn infants in low-dose group had significantly better outcomes in all three domains of the neurobehavioral test.

**Conclusion:**

7 mg/kg STP is superior to 5 mg/kg in creating deeper hypnosis for mothers. However, it negatively impacts Apgar score and neurobehavioral test of neonates. STP seems to has dropped behind as an acceptable anesthetic in Cesarean section.

**Trial registration:**

IRCT No: 2016082819470 N45, 13/03/2019.

## Introduction

Determining the optimal dosage of anesthetic agents is challenging. This fact is particularly a matter of concern in Caesarean section [1, 2]. The susceptible fetus can be affected by the administered agents passing through the placenta, resulting in the delivery of anesthetized “sleepy baby” [3]. Robust study on appropriate drug regimens to guarantee adequate depth of anesthesia during Caesarean section is surprisingly rare. This may be due to paucity of use of general anesthesia for Caesarean section and its application only in emergency situations when conducting randomized trials is extremely difficult.

Sodium thiopental (STP), a short-acting well known barbiturate, is currently a routine choices for induction of general anesthesia in Cesarean section in some countries [4]. The usual recommended dose of thiopental for induction of general anesthesia for Caesarean section is 4–5 mg/kg, but several studies showed that parturient are at risk of inadequate anesthesia [5]. The incidence of unexpected awareness during Caesarean has been decreased to 0.26–0.4% by using modification of induction technique and larger dose of thiopental, but it is still more prevalent than in general surgical population (0.1–0.2%) [6, 7]. Obstetric general anesthesia includes many risk factors for accidental awareness during general Anesthesia (AAGA) including use of STP for anesthesia, rapid sequence induction, deep neuromuscular block, obesity, difficult airway management, and emergency surgery [8]. Thiopental in combination with rapid sequence induction is an important risk factor for awareness, possibly because of inappropriate low dose [8].

The bispectral index (BIS) is a sensitive objective tool which analyses the patient’s electroencephalogram (EEG) and represents a 0 (silence) to 100 (complete wakefulness) scale. Values ranging from 40 to 60 indicate appropriate hypnosis for surgery [9–11]. However the isolated forearm technique (IFT) has been proposed as the gold standard test for detecting wakefulness during Caesarean section [12]. It is based on isolation of the forearm from the effects of neuromuscular blocking drug by occlusion of the circulation by a pneumatic tourniquet inflated before injection of neuromuscular blocking agent. Movement of the hand in response to a recorded command played to the patient is then monitored [12, 13]. Nevertheless, it has been reported that lower than previously recommended values for BIS are needed to avoid IFT test responses during laryngoscopy, intubation and skin incision [14]. Some investigators have reported that despite a median BIS of less than 70 (range of 42–68) on all parts of general anesthesia in Caesarean section, hemodynamic parameters increased significantly in some patients especially during laryngoscopy and intubation, where routine dose of 4–5 mg/kg thiopental dose was used [5].

Although thiopental dose of 5–7 mg/kg has been described safe for induction of anesthesia in Caesarean section [4, 15], the dosage of medication should be adjusted so that the mother can benefit from satisfactory anesthesia, while the safety of the fetus in provided as well. We designed this randomized clinical trial to compare the effects of higher versus lower doses of STP on the depth of anesthesia with IFT and BIS (primary outcome) in the parturient and its side effects measured by Apgar score and neurobehavioral test (secondary outcomes) in the newborns immediately after delivery.

## Material and methods

This single blind randomized clinical trial was registered in Iranian Randomized Clinical Trial Registry (IRCT No: IRCT2016082819470N45, 13/03/2019), conducted in pregnant women with American Society of anesthesiologist (ASA) physical status I, II score scheduled for elective Cesarean section in Hafez hospital. The study protocol was approved by Ethics Committee of Shiraz University of Medical Sciences. Exclusion criteria were regional anesthesia, neuromuscular and psychiatric disorders, history of awareness in previous anesthesia, opioid dependent patients, receiving magnesium sulfate, anti-psychotic and anti-hypertensive medications, predicted need to vasopressor or vasodilator agents during surgery, poor cooperation and women with known fetal problem.

An expert anesthetist informed the eligible parturient about the choice of general and spinal anesthesia and their advantages and disadvantages. The anesthetist also fully explained to the parturient the research steps and written consent form was filled out by the patients.

The sample size calculation was performed according to our previous study on sodium thiopental 5 mg/kg [11], and a pilot study on thiopental 7 mg/kg, that the between-group difference in incidence of inadequate depth of anesthesia by IFT test was 25% approximately. By calculating type 1 error of 5%, power of 80%, and drop-out rate of 10%, each group required 40 patients.

After enrollment, the participants were randomized into low-dose (5 mg/kg) or high-dose (7 mg/kg) STP groups. For allocating the patients into the intervention and control groups, according to research randomizer site (http://www.randomizer.org), random numbers were produced and two custom-built sets of random numbers were generated and kept in a sealed envelopes. Then the patients were allocated into one of two groups by an independent individual before induction of general anesthesia. The patients, anesthetist, and the two independent observers who documented the BIS and IFT scores were blinded to the group allocations. In addition, as monitoring of depth of anesthesia is not routine in our hospital daily practice, the anesthetist was blind to BIS. Generating the random allocation sequence, measurements, assigning participants to interventions were done by individuals who were blinded to study.

All patients were monitored using routine noninvasive blood pressure, electrocardiography, pulse oximetry, end tidal gas analyzer and BIS monitoring. After proper pre oxygenation, general anesthesia was induced with rapid sequence method and administration of either 5 mg/kg STP or 7 mg/kg STP and 2 mg/kg succinylcholine. Maintenance was accomplished using 50% O_2_, 50% N_2_O and sevoflurane was titrated based on the end tidal concentration to keep it between 1.8–2.2% before delivery of the fetus thereafter, it was adjusted to about 1.2%. After delivery of the neonate, 0.15 mg/kg morphine and 0.02 mg/kg midazolam were administrated. After return of spontaneous respiration, 0.3 mg/kg atracurium was administered to provide surgical relaxation. Sevoflurane and N_2_O were discontinued at the time of subcutaneous and skin suturing, respectively.

The examiner explained the concept of the study to the patients and placed a pneumatic tourniquet around the right forearm of the patients and inflated it to 200 mg immediately before induction. After induction, a recorded message was played by the earphones every 1 min which asked the patient to move the fingers of her right hand. Hand activity was scored as no movement (0), non-specific movement (e.g. fine movements of fingers) [1], or firm clenching/flexing movement. The BIS value, IFT response and end tidal sevoflurane concentration were documented during the following events: baseline, anesthesia induction laryngoscopy, intubation, skin-peritoneal-uterine incisions, uterus retraction, delivery, uterine closure, muscular closure, subcutaneous closure, skin closure, sevoflurane discontinuation, eye opening and tracheal extubation.

A trained examiner asked the patients five questions, 12–24 h after surgery, about any experience of dreaming or recall during the anesthesia and surgery. The patients were asked: “What was the last thing you remember before going to sleep?” What was the first thing you remember when you woke up?” Can you recall anything between?” and “Did you have any dreams during your anesthetic?” [16, 17]. Apgar scores of the newborn infants were measured at 1, 5 and 20 min after delivery. Neurobehavioral test was performed 20 min after delivery. A quantitative rather than qualitative assessment of neonatal neurobehavioral status would be valuable in the identification of infants at risk for developmental disabilities. After introduction of Brazelton on Neonate Behavioral Assessment Scale (BNBAS) in 1973, Morgan A et al. designed and standardized a new assessment scale that would assess the various aspects of neurobehavioral fitness at a given conceptional age [18]. It consists of 27 items divided into three sections.

1. Tone and motor patterns
2. Primitive reflexes
3. Behavioral responses.

Each section consists of items scored on a three-point scale [18]. A trained midwife who evaluated the APGAR score and neurobehavioral test was blinded to the mother’s study group allocation.

The primary outcomes were mother’s IFT and BIS as measures of depth of anesthesia. The secondary outcomes were newborns’ Apgar score and neurobehavioral test results.

The data were evaluated by SPSS 20 software (SPSS Inc., Chicago, Il). Normality was assessed by Kolmogorov-Smirnov test and the obtained quantitative data were analyzed using Mann-Whitney and repeated measurement test, and the qualitative data analysis was done by Chi-square and Fisher exact test. A two sided *P* value of less than 0.05 was considered statistically significant.

## Results

**Fig. 1:**
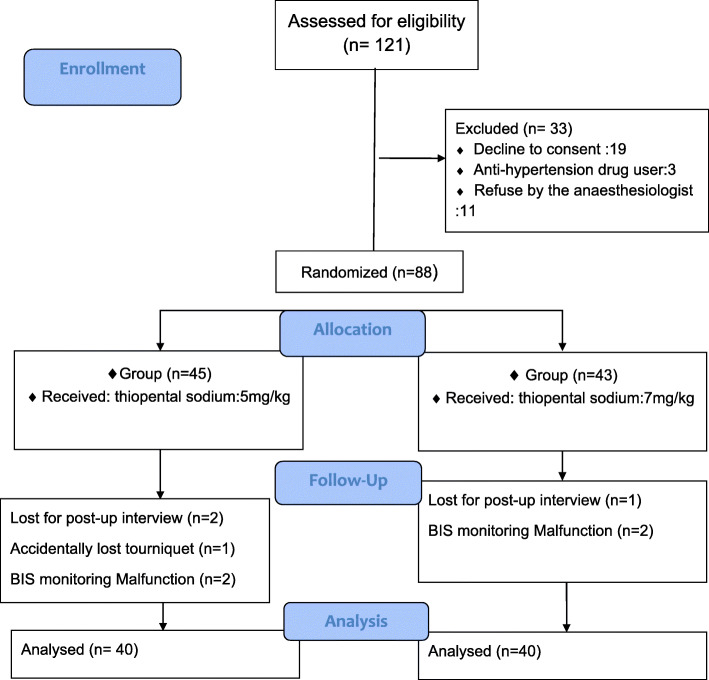
Consort flow chart. Out of 121 patients who were screened for eligibility criteria, 33 patients were excluded and 8 patients were lost during data gathering. Thus 40 patients in each group were analyzed

The study was performed from August to November 2018. Out of 121 patients who were screened for eligibility criteria, 33 patients were excluded (Fig. 1) and 8 patients were lost during data gathering (5 of them were in the group of 5 mg/kg of STP and 3 were in the group of 7 mg/kg STP, no unwanted event was observed in these 8 cases). Thus 40 patients were in each group. One parturient in the low dose STP group had twin pregnancy. There were no significant differences regarding demographic data of patients including age, weight, and duration of anesthesia, surgery and surgery to delivery time in baseline characteristics of two groups (Table 1). BIS was significantly lower in high-dose group in the time interval between skin incision to delivery 36.86 ± 4.37 **vs** 39.74 ± 6.83 (*P*-value = 0.02), as well as at the point of subcutaneous closure 42.77 ± 2.57 vs 45.09 ± 4.33 (*P*-value = 0.03) and skin closure 49.50 ± 3.91 vs 52.39 ± 4.28 (*P*-value = 0.04) (Table 2).

**Table 1:** Baseline demographic and operation characteristics of 80 pregnant women undergoing caesarian section

Parameters	5 mg/kg	7 mg/kg	***P***-value
Age (years)	30.3 ± 4.7	29.90 ± 5.6	0.79
Weight (kg)	70.1 ± 9.7	67.6 ± 6.4	0.18
Surgery duration (minutes)	70.8 ± 8.2	71.2 ± 8.8	0.87
Anesthesia duration (minutes)	90.2 ± 10.9	90.9 ± 12.1	0.79
Duration of anesthesia to surgery (seconds)	104.3 ± 10.6	105.8 ± 2	0.80
Surgery to delivery (minutes)	6.6 ± 1.7	6.8 ± 1.1	0.47

**Table 2:** Bispectral index (BIS) of 80 pregnant women (40 = low-dose group and 40 = high-dose group) undergoing caesarian section

Event	5 mg/kg	7 mg/kg	***P***-value
Baseline	95.97 ± 1.56	95.55 ± 1.61	0.21
Induction to skin incision	43.89 ± 4.87	42.81 ± 3.88	0.38
Skin incision to delivery	39.74 ± 6.83	36.86 ± 4.37	0.02
Delivery to Extubation			
Uterine closure	34.75 ± 2.33	36.02 ± 3.36	0.23
Muscular closure	40.46 ± 3.85	38.57 ± 3.21	0.36
Subcutaneous closure	45.09 ± 4.33	42.77 ± 2.57	0.03
Skin closure	52.39 ± 4.28	49.50 ± 3.91	0.04
Stop volatile	60.60 ± 3.66	59.80 ± 5.27	0.62
Eye opening	73.12 ± 5.88	72.52 ± 6.41	0.91
Extubation	83.85 ± 3.43	82.37 ± 3.44	0.06

Induction to skin incision: induction, laryngoscopy, intubation

Skin incision to delivery: skin incision, peritoneal incision, uterus incision, uterus retraction, delivery

The IFT values for induction, laryngoscopy and intubation stages were combined to give 120 data points (intubation to skin incision). The IFT values for skin incision, peritoneal incision, uterus incision, uterus retraction and delivery stages were combined to give 200 data points (skin incision to delivery). The IFT values for uterus closure, muscular closure, skin closure, subcutaneous closure, stop volatile, eye opening and extubation were also combined to give 280 data points (Delivery to extubation) (Table 3). Significant differences were noticed in IFT scores between two groups in induction to incision and skin incision to delivery stages (Table 3). None of the patients recalled dreaming experiences during the course of surgery when asked during the postoperative interview.

**Table 3:** IFT of 80 pregnant women (40 = low-dose group and 40 = high-dose group) undergoing caesarian section

Event	IFT = 0		IFT = 1		IFT = 2		***P***-value
	5 mg/kg	7 mg/kg	5 mg/kg	7 mg/kg	5 mg/kg	7 mg/kg	
Baseline (*n* = 40)	0	0	0	0	40	40	> 0.999
Induction to skin incision (*n* = 120)	62	69	42	39	16	12	0.04
Skin incision to delivery (*n* = 200)	190	195	9	4	1	1	0.03
Delivery to extubation (*n* = 280)	247	244	30	32	3	4	0.27

*IFT* Isolated forearm technique

Induction to skin incision: The IFT values for induction, laryngoscopy, and intubation were combined to give 120 data points

Skin incision to delivery: The IFT values for skin incision, peritoneal incision, uterus incision, uterus retraction, delivery were combined to give 200 data points

Delivery to extubation: The IFT values for uterus closure, muscular closure, skin closure, subcutaneous closure, stop volatile, eye opening, extubation were combined to give 280 data points

**Fig. 2:**
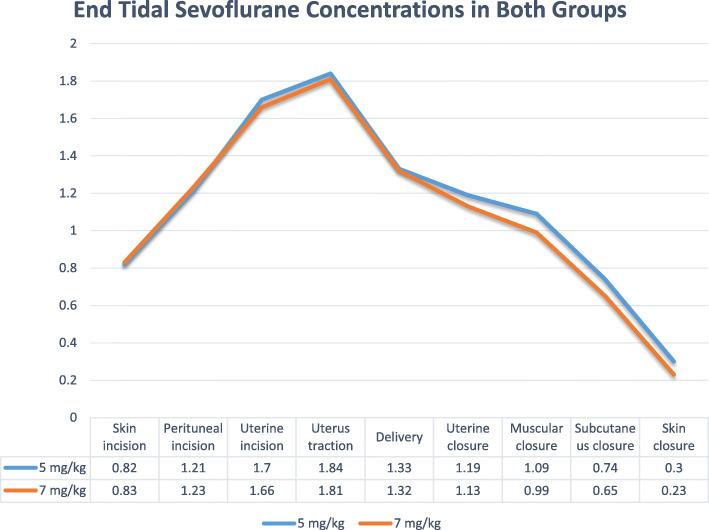
End tidal Sevoflurane concentrations at different phases of surgery. End tidal sevoflurane concentration peaked at the point of uterine traction in both groups, the differences were significant for the closure of uterus (*P*-value = 0.046), subcutaneous tissue (*P*-value = 0.036), and skin closure (*P*-value = 0.046)

Apgar scores showed only a significant difference at minute 1 after delivery (*P*-value< 0.001). 47.5% of participant in a high dose STP group and 5% of participant in a low dose STP group had Apgar score below 7 at minute 1 after delivery that was a significant difference (*P*-value< 0.001). However, all the participant in both group had Apgar score > 7 at 5 min after delivery (Table 4). Newborn infants from low-dose group showed significantly better outcome in all three parts of neurobehavioral test (Table 5). End tidal sevoflurane concentration peaked at the point of uterine traction in both groups, the differences were significant for the closure of uterus (*P*-value = 0.046), subcutaneous tissue (*P*-value = 0.036), and skin closure (*P*-value = 0.046) (Fig. 2).

**Table 4:** Apgar scores of 81 newborn infants at 1, 5 and 20 min after delivery

Minute		5 mg/kg	7 mg/kg	***P***-value
1 min after delivery	Mean ± SD	8.73 ± 1.24	7.82 ± 0.87	< 0.001
	≤7: n(%)	2 (5%)	19 (47.5%)	< 0.001
	> 7: n (%)	38 (95%)	21 (52.5%)	< 0.001
5 min after delivery	Mean ± SD	8.87 ± 0.89	8.90 ± 0.84	0.91
	≤7: n(%)	40 (100%)	40 (100%)	1
	> 7: n (%)	40 (100%)	40 (100%)	1

**Table 5:** Neurobehavioral test of 81 newborn infants at 1, 5 and 20 min after delivery

Parameters	5 mg/kg (***n*** = 369)			7 mg/kg (***n*** = 360)			***P***-value
	1 min after delivery	5 min after delivery	20 min after delivery	1 min after delivery	5 min after delivery	20 min after delivery	
Tone and motor patterns	1	36	332	3	55	302	0.04
Primitive reflexes	1	10	358	3	22	335	0.04
Behavioral responses	1	13	355	5	24	331	0.03

## Discussion

We studied the different aspects of adequacy and safety of two STP doses in general anesthesia for cesarean section. Based on our findings, 7 mg/kg STP is superior to 5 mg/kg in creating deeper hypnosis in the parturient scheduled for elective Cesarean section under general anesthesia, However, it negatively impacts Apgar score and neurobehavioral test of neonates.

In our patients, BIS scores were not significantly different prior to skin incision. This can be attributed to the fact that the medication has not reached the maximum level in this phase. In contrast, lower BIS in the high-dose group in the time interval from skin incision to delivery was clearly significant, which shows a greater depth of anesthesia. It is noted that skin incision creates a great stimulus [5]. As stimulations increase from skin incision to delivery, the responses on BIS are more amplified. Although BIS is in acceptable range in both groups (40–60), this level of BIS could not prevent IFT test response during anesthesia stages (Table 3). Therefore, it seems that BIS is not a completely reliable index to monitor the depth of anesthesia in these phases. In addition, IFT showed significant differences in both inductions to skin incision and skin incision to delivery periods. The patients in the high-dose group had less frequent hand movements, which indicate deeper hypnosis.

Overall, both BIS and IFT tests showed a deeper level of anesthesia in high-dose group during the course of skin incision to delivery. Nonetheless, this finding was not similar for induction to skin incision period. While IFT showed a deeper anesthesia in high-dose group, BIS failed to show any significant difference. The inefficiency of BIS to differentiate between positive and negative IFT in early stages of Cesarean section was mentioned by Zand et al. [14] and Russel et al. [13] as well. It can be concluded that IFT is more reliable in this regard. This is also in accordance to the fact that no patient had recall and memory of events in our patient population. The apparent unresponsiveness of the patient should not be mistaken for unconsciousness [19]. As a result, there are several arguments questioning the value of IFT; however, the advantages make its utility reasonable [20].

Although 1-min Apgar score indicates the requirements for neonate cares at the time of birth, this is the 5-min Apgar score that shows the morbidity and the effect on the neurobehavioral response [21, 22]. In our study we found that the neonates had lower Apgar score at minute 1 in high-dose group and subsequently improved at 5 and 20 min. Thus for better evaluation of the neonatal developmental disability we used neonatal neurobehavioral examination. Low-dose STP group neonates performed better in all the three aspects of tone and motor patterns, primitive reflexes, and behavioral responses for neurobehavioral test. This is in line with the results of minute 1 Apgar score and indicates that the 5 mg/kg dosage is relatively safer for infants. If an adequate anesthesia depth was accomplished with 5 mg/kg thiopental sodium, administration of higher dosage would not be advisable. However, it was demonstrated in the study that 5 mg/kg dose may be associated by lower BIS scores and more positive IFT tests, although these undesirable observations were not translated to apparent awareness of the patients during post-operative interview [23].

Other alternative intravenous anesthetics are advised for induction of anesthesia in Caesarean section such as propofol [24]. Some studies stated that propofol and thiopental do not have a significantly different influence on the Apgar score, while propofol makes deeper anesthesia, shorter recovery time, better hemodynamics and prepares appropriate uterine relaxation during fetal delivery [11, 25–30]. Induction with propofol also results in a significantly lower umbilical arterial oxygen saturation than induction with thiopental,but multiple trials indicates that propofol and thiopental are equally suited for Caesarean section [31]. However, some side effects such as propofol induced pain on injection and sever bradychardia when combined with succinylcholine for rapid-sequence induction makes some anesthesiologists reluctant in its use [32]. This reluctance is especially realizable where other rapid acting muscle relaxants like rocuronium is not readily available.

In conclusion, 7 mg/kg STP is superior to 5 mg/kg in creating appropriate hypnosis for induction of general anesthesia for cesarean section. However, it negatively impacts Apgar score and neurobehavioral test of newborn during early phase of birth. An acceptable intravenous anesthesia should be safe for the neonates while providing acceptable depth of anesthesia for the parturient. Therefore, STP couldn’t be recommended as an ideal medication for induction of general anesthesia in Cesarean section anymore.

## Data Availability

All data will be available on request.
